# Metric-Based Simulation Training to Proficiency for Endovascular Thrombectomy in Ischemic Stroke

**DOI:** 10.3389/fneur.2022.742263

**Published:** 2022-09-27

**Authors:** Markus Holtmannspötter, Robert A. Crossley, Thomas Liebig, Anthony G. Gallagher

**Affiliations:** ^1^Department of Neuroradiology, Nuremberg General Hospital, Paracelsus Medical University, Nuremberg, Germany; ^2^North Bristol National Health Service (NHS) Trust, Bristol, United Kingdom; ^3^Department of Neuroradiology, Ludwig-Maximilians-University (LMU) Munich University Hospital, Munich, Germany; ^4^Faculty of Medicine, Katholieke Universiteit (KU) Leuven, Leuven, Belgium; ^5^Orsi Academy, Melle, Belgium; ^6^School of Medicine, Faculty of Life and Health Sciences, University of Ulster, Coleraine, United Kingdom

**Keywords:** proficiency-based progression, metric-based, endovascular thrombectomy (EVT), simulation training, virtual reality simulation

## Abstract

Ischemic stroke is one of the leading causes of death and long-term disability in the West. Mechanical revascularization techniques are considered the standard of care for large vessel occlusive stroke. Traditional apprenticeship models involve doctors training their skills on patients. Simulation platforms have long been recognized as an alternative to this. There has however been very little robust assessment of the training outcomes achieved on some of these platforms. At best, these simulations increase understanding of the procedural process and may help improve some technical skills; at worst they may instill bad habits and poor technique. The prerequisite of any simulation process must be to teach what to do, with which devices, in the correct sequence as well as what *not* to do. It should provide valid metric-based feedback to the trainee that is objective, transparent, and fair for formative and summative performance feedback. It should deliver a training program that measures the performance progress of trainees against expert benchmarks—benchmarks that represent an evidence-based peer-reviewed standard. In this paper, we present a perspective for PBP training for thrombectomy based on our experience with the process of procedure characterization, metric validation, and early experience of using this approach for proficiency training. Patient outcomes are not only determined by optimal performance in the Angio Suite but also by an efficient patient procedure pathway. There will be value in utilizing the PBP training standard not only for the procedure itself but also for the constituent elements of the stroke pathway to further improve treatment outcomes for ischemic stroke patients.

## Background

Acute stroke is a common and devastating condition that causes the death of one-third of patients within 6 months and leaves another third permanently disabled. Prospective and randomized clinical trials on mechanical thrombectomy for large vessel occlusions have led to a revolution in treating ischemic stroke patients. Its efficacy is unmatched by any previous therapy in stroke medicine. Despite the proven effectiveness of mechanical thrombectomy, access to this treatment is limited in many countries, in part due to the lack of specially trained doctors.

## Agents of Change

Changing work practices and the evolution of more complex interventions in surgery, interventional radiology, cardiology, and medicine are forcing a paradigm shift in the way doctors are trained (1). Minimally invasive surgery (2), implantable cardioverter defibrillators (ICDs), cardiac resynchronization therapy (CRT) (3), transcatheter aortic valve implantation (TAVI) (4), and acute stroke intervention procedures (5, 6) are producing these changes at a faster pace than in other medical disciplines. Consequently, surgery, radiology, and cardiovascular medicine have had to develop a sophisticated understanding of precisely what is meant by “training” and “skill”. This understanding is derived from psychological science, and the main findings have been generated from a quantitative applied experimental psychological approach (based on metrics) (7, 8). However, these need to be transferred into clinical practice requiring customized adaptation by the respective clinical disciplines themselves (translational science).

In the US, the 2014 report from the Institute of Medicine, Committee on the Governance and Financing of Graduate Medical Education that meets the nation's health needs came with a stark message: training in medicine must move to “outcome” rather than “process” driven graduate medical education (GME) (9). The settled conclusion is that deliberate practice (10) training on a (virtual reality) simulation presents the best current solution. These simulations should characterize the important performance characteristics of procedural skills which have been derived and operationally defined from, and then benchmarked by experienced surgeons (i.e., level of proficiency). Simulation training is optimal with metric-based feedback, particularly formative assessments on trainee procedural error enactment, proximate to their performance. In prospective, randomized studies (11–13), learners trained to a benchmarked proficiency level on the simulator performed significantly (i.e., 15–60%) (14) better than learners who were traditionally trained. Endovascular medicine has the most sophisticated virtual reality simulators available in medicine, and these have been used for the rollout of interventions such as carotid artery stenting in the US. The US Food and Drug Administration (FDA) has advocated the use of simulations as part of the approval of new devices (15, 16) and the American Board of Internal Medicine has adopted simulation as part of the maintenance of certification (17). Simulation is rapidly becoming a mainstay of surgery and endovascular education, training, certification, and the safe adoption of new technology.

## A Scientific Approach to Training

Although the methodologies to act on a more efficient and effective approach to learning skills exist, their implementation is not trivial. Simulation is useful, but without the proficiency-based progression (PBP) process, it is not as effective. PBP puts a clear measurable structure to simple simulation and gives clear feedback to the trainee. PBP (8) training on simulations outside the clinical environment can augment and quality-assure a work-based approach to skill acquisition. Considerable validation evidence already exists as to the effectiveness of this approach to training clinical skills (14, 18). However, PBP training programs require detailed, comprehensive, and validated metric-based characterization of the skills to be learned (19). Such metrics are also used to establish a quantitative benchmark that trainees must demonstrate before training progression or completion. Benchmarks are derived from, validated by, and benchmarked based on the objectively assessed performance of experienced clinicians. Trainees are fully cognizant of the metrics which are also used to implement a deliberate practice (10) rather than a repeated practice approach to training. Training is complete only on demonstration of the proficiency benchmark. When applied with scientific rigor, a PBP approach to learning skills is very effective, objective, transparent, and fair to the trainee and the training organization.

The PBP approach to training is based on solid research and has been validated in different healthcare settings for over a decade [e.g., laparoscopic (11–13), arthroscopic (20, 21), robotic (22), endovascular skills (23), anesthetic (24–26), mechanical thrombectomy for ischemic stroke (5), and communication skills (27)]. Results from the first multicenter randomized prospective trial of proficiency-based progression simulation training (for an arthroscopic shoulder procedure) showed that intra-operative errors were reduced by 56% when compared to the standard approach to training (21). The magnitude of the reduction in epidural analgesia failure during labor was similar (i.e., 53%) (28). Those randomized trial results demonstrate that requiring trainees (no matter how senior or experienced) to “train” and use a simulation or skills laboratory does not guarantee quality assurance and verified performance level at the completion of training. On the other hand, using the exact same resources with a PBP curriculum and the requirement to demonstrate quantitatively defined skills benchmarks does. The first step in using this approach is to develop and validate the performance metrics which characterize optimum (and sub-optimal) procedure performance. Once validated the metrics can then be used to guide the construction of a PBP training curriculum and the establishment of proficiency benchmarks that trainees must demonstrate before successful training completion and progression to the implementation of their skills in a clinical setting.

## PBP Methods

**Figure 1:**
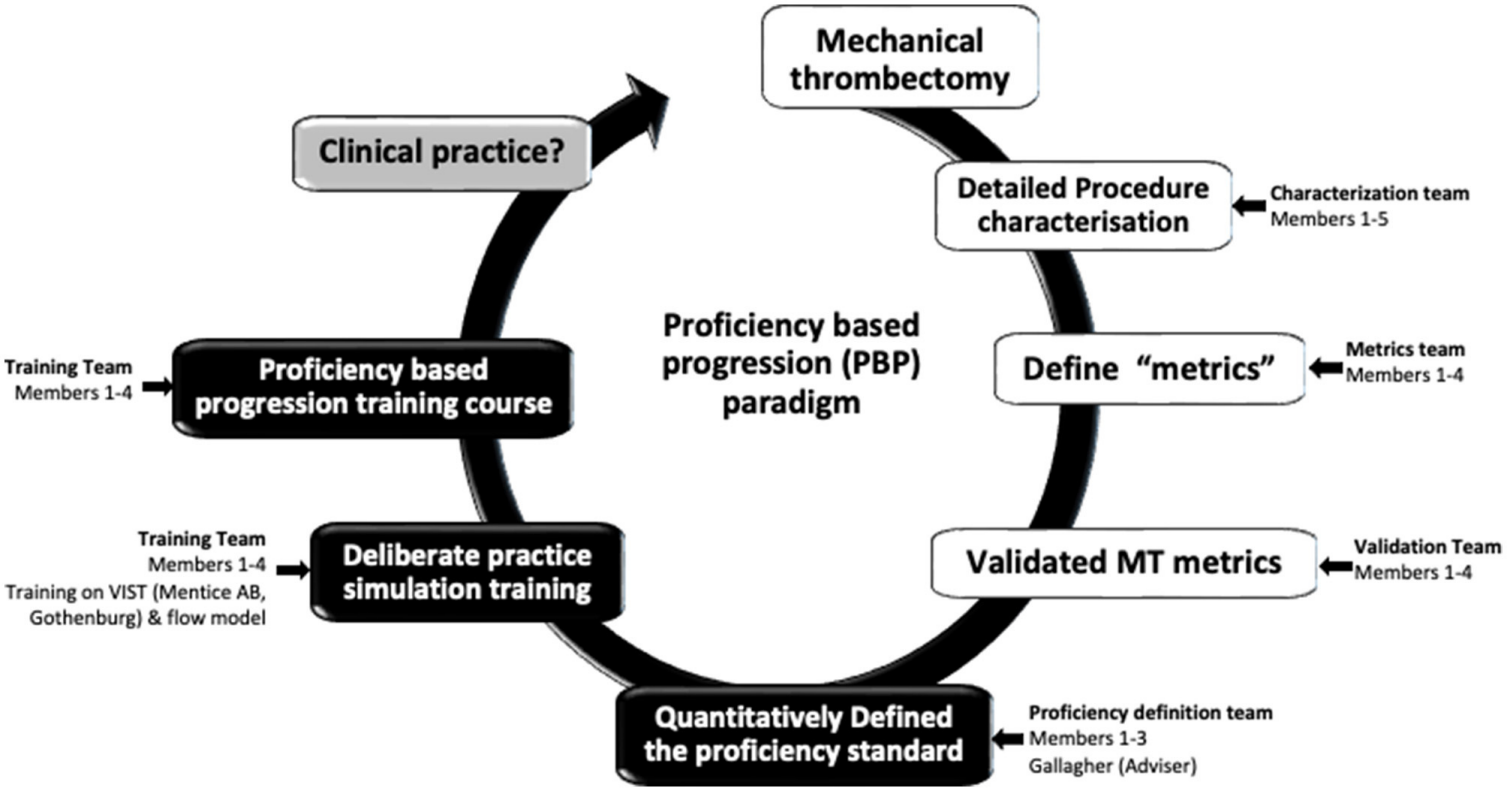
The Proficiency based progression (PBP) metric development, validation and training paradigm (Crossley,^1^ Holtmansspötter,^2^ Liebig,^3^ (Interventional Neuro Radiologists); Gallagher^4^ (Behavioural Scientist); Lindkvist^5^ (VR Sim engineer advisor)).

During preliminary meetings of the team (Figure 1) consensus was reached to comprehensively characterize a “reference approach” (i.e., straightforward, uncomplicated, commonly encountered, accepted, etc.) to the performance of the procedure e.g., mechanical thrombectomy (MT). The team then set about identifying the procedure-phases, steps, errors, and critical errors. This meant that the investigators identify and then operationally define these behavioral units, i.e.,

- the steps required to perform the procedure safely
- the performance characteristics that indicate deviations from optimal performance (or errors)
- fundamental performance errors (or critical errors) which expose the patient or operator to unnecessary risk

For the MT procedure, these goals are facilitated by viewing video recordings of the procedural performance (5, 20, 29). Viewing was initially done by the investigators in the same room with ongoing verbalized descriptions of performance and interaction between the investigators about what they were viewing, its meaning, and whether the performance was as per “instruction for use”, optimal or sub-optimal.

## Metrics Stress Testing and Definition Verification

**Figure 2:**
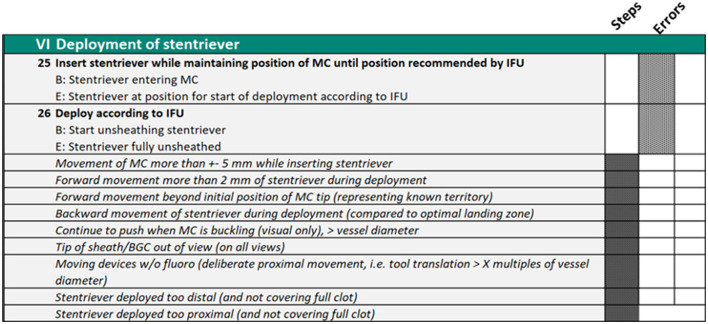
Phase VI of the procedure metrics which explicitly define what the operator must do to complete the procedure and the errors they should avoid.

When the team was satisfied that they had characterized a reference approach to the procedure in its entirety, they began the process of metric verification as operationally defined (8, 19, 30). This process involved the scoring of novel video recordings of the procedures. The team scored these video recordings initially all at the same time but latterly in discrete pairs. The function of these scoring exercises was to stress test the applied and practical usage of the metrics and their operational definitions. Problems with either of these aspects were usually indicated by low inter-rater reliability of scores. Metrics that are not scored reliably would need to be redefined or removed from the scoring matrix (see Figure 2 as an example for some metrics).

## Face and Content Validity Assessment—Delphi Meeting

Once the metrics (that were demonstrated as being representative of the procedure to be characterized and could be reliably scored) were identified, they were presented to an independent group of peers during a ~3-h face-to-face meeting (5, 20, 29, 31). This group of individuals was selected because they had very good knowledge of the endovascular thrombectomy procedures that had been characterized and they were also judged to be independent and fair-minded. Their task was to reach a consensus on whether the metrics and their operational definitions appropriately characterize the reference procedure in question. On the basis of consensus, metrics or groups of metrics were accepted, modified, or rejected. A very high level of consensus could be achieved (5).

## Assessment of Construct Validity

Metrics retained as part of the procedure characterization and agreed by the Delphi meeting were then used to establish the construct validity, i.e., the metrics distinguish between the objectively assessed performance of experienced and novice interventionalists when performing the procedure. After an initial period of assessor training to achieve inter-rater reliability >0.8 of metric identification between raters, objective assessment of novel videos by pairs of raters commenced. If valid, the metrics should demonstrate a significant difference in scores between experienced and novice operators (32, 33). In the mechanical thrombectomy project the metrics demonstrated good construct validity (5).

## Proficiency Definition

On demonstration of construct validity for the metrics, the team met to reach a consensus on which metrics or groups of metrics' proficiency should be defined. This involved the metrics which best or most reliably distinguished between experienced and novice operators. It also involved the identification of performance characteristics which were a compulsory part of proficiency demonstration. The proficiency benchmark was thus quantitatively defined (19, 21, 23).

## Proficiency vs. Competency

While some clinicians are concerned that perhaps the skill level for PBP training has been established at too high a benchmark for trainees, experience suggests that most trainees will reach this level. In a recent study, Angelo et al. demonstrated that >80% of trainees demonstrated the proficiency benchmark on a weekend course for learning two arthroscopic procedures (34). Furthermore, it has also been demonstrated that trainees, who had been allowed to train on the proficiency-based progression training program but had not reached the requisite level of proficiency, performed better than their traditionally trained peers, but markedly less well than those who did demonstrate proficiency at the termination of training (13). The advantage of a proficiency-based progression training program is that it is transparent, objective, and fair. Furthermore, it is flexible enough to deal with individuals who acquire their skills at a slower rate just as easily as those who acquire their skills more quickly. Also, this approach to training ensures a less variable graduating skill level. Contrary to popular belief, the developers of this approach to training do not assume that a trainee who has acquired the mean technical performance capability of practicing surgeons, has acquired the same level of wisdom. The goal has simply been to ensure a skill level of the trainee that indicates that their procedural performance has been automated to the point where they have the attentional capacity to hear and to follow instructions from the master surgeon/physician/radiologist during an intra-operative training procedure (8), an interpretation which has recently been validated (35). Proficiency-based progression training ensures that the learning experience in the operating room is more efficient and effective, thus operative procedures that the trainee is exposed to are used for maximal learning benefit. This approach to training does not presume some binary acquisition of technical skills or knowledge or decision making (36), rather skill acquisition is seen as a developmental process. All trainees using this approach are first taught anatomy and physiology, what to do and what not to do, *but* are not allowed to proceed to *in vivo* training before demonstrating the requisite knowledge (11). This ensures that training goals can be accomplished more effectively and efficiently, and quality assures the progression process.

## PBP Course(s) for Mechanical Thrombectomy

Courses were delivered at the ASSERT center (UCC, Cork, Ireland), a bespoke simulation facility, over a 2-day period. Interventional Neuroradiology Fellows from the UK, Belgium, and the Netherlands of varying experience were invited to attend. The minimum skill set required was to be (as judged by their department) “competent in cerebral angiography.”

The course introduction involved outlining the concepts of PBP and a description of the reference approach. A demonstration case was then performed by faculty members with commentary to demonstrate phases, steps, and errors in the procedure.

Delegates were then given time to familiarize themselves with the model before being asked to perform an unprompted case. Faculty assisted with the operation of the simulator but did not offer technical procedural assistance. This formed the baseline delegate performance assessment.

**Figure 3:**
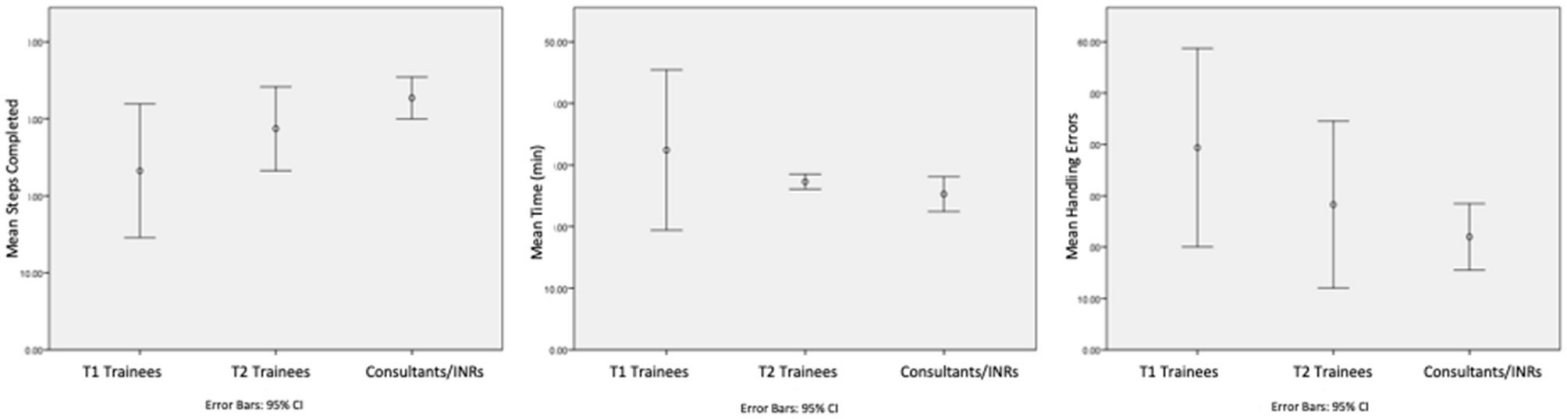
T1 represents trainees at baseline, T2 represents trainees after the course and Consultants/INRs represent the proficiency benchmark. The performance of the trainees in terms of number of procedure steps completed, procedure time and handling errors all improved, but none of them reached the proficiency in the given time of the course.

In a series of small group practical workshops, the delegates were given intensive training on the procedure with immediate proximate feedback on performance. At the end of the course, a further assessment case was performed in the same manner as the baseline case had been, with faculty providing technical simulator assistance but no procedural input. An overview of the results from these early courses is summarized in Figure 3.

Performance in terms of steps and time taken became more homogenous among the trainees; this increasing homogeneity of performance is an indicator but not a guarantor of increasing skill.

It is also important to note, that despite improvements being made none of the trainees achieved the performance benchmark. In essence, the platform was still able to discern the performance of those in training vs. experts. Fundamentally, a 2-day simulation course does not replace clinical training and experience.

## Training MT Skilled Performance

Mechanical thrombectomy is a life-changing procedure for patients but it is a high-risk procedure for the clinician to perform and requires considerable skill. Traditionally these skills have and still are widely acquired through the apprenticeship-style model on real patients over an extended period of training, like many other similar surgical and interventional skills. Without starting to train directly on patients, physics-based virtual reality simulation offers the potential solution to this considerable problem. Used properly, VR simulation ensures that the trainees, regardless of seniority, do not perform the procedure until they are skilled enough (i.e., proficiency-based progression). As demonstrated in other specialties in prospective, randomized, and blinded clinical trials, a proficiency-based progression approach to learning to perform the procedure utilizing VR simulation, is safer, more effective, and efficient. It ensures that the trainee knows what to do and what not to do before they attend the skills laboratory for training. Customizing this for mechanical thrombectomy, trainees are taught in the skill laboratory how to do the procedure on a physics-based VR simulation by intervention neuroradiologists, who are very experienced and good at performing the procedure and know the metrics. Faculty need specific training on the metrics as it is the VR simulation and their capability to deliver timely, explicit, constructive, and formative feedback to the trainee that determines training effectiveness. Trainees must know that they will not progress in their training until they demonstrate the requisite proficiency benchmarks.

## Patient Outcomes

Though it has not yet been proven in mechanical thrombectomy, there is a growing body of evidence from other areas of medicine and surgery that demonstrates that skill of the surgeon is linked to patient outcomes (37) including e.g., cancer procedures (37). It has been demonstrated that a PBP training program significantly reduces failure rates e.g., in epidural anesthesia procedures (28). What is still not well-understood is the specifics of operator performance and how they impact patient outcomes. In mechanical thrombectomy, patient outcomes are not only determined by optimal performance in the Angio Suite but also by an efficient and effective patient procedure pathway. Delays in the treatment pathway due to sub-optimal infrastructure or communications are known to contribute to some patients' poor outcomes. A 30 min delay in reperfusion, regardless of the means, reduces the chance for a neurological independent outcome by 10 %, Kathri et al. analyzed on basis of IMS III data (38).

The striking example of simulation training for thrombolysis treatment in Stavanger, Norway underlines that pathway time savings can be greatly enhanced by simulation training too. For instance, reducing the door-to-needle time from 30 min down to 13 min (39). We propose the value of utilizing proficiency-based training standards in the constituent elements of the stroke pathway before and after the Angio Suite to further improve treatment outcome results for ischemic stroke patients, thereby broadening the use of simulation with PBP not confined to the procedure alone. We foresee benefits in pre-hospital, imaging assessment, and transport/infrastructural aspects of regional pathways. It will not require every constituent step in the pathway to be reinvented but will likely lead to greater structured interconnectivity and parallel simultaneous decision making. Challenges remain to integrate different disciplines at regional and local levels. Different health systems will face different infrastructure, population, and geographical challenges, and it is likely that a system that works in Southern Germany may not be exactly the same as one that works in Southwest England. However, there will be themes and common principles that represent “exemplar” practice in any location.

Dave Brailsford attributed the phenomenal success of the British Olympic cycling team at the London 2012 Olympic Games to the “aggregation of marginal gains” (AMG). AMG, he explained, is “the 1% margin for improvement in everything you do”. He described this process of multiple and seemingly minuscule improvements throughout the athlete's entire preparation process for competition, which, collectively achieved a far superior track performance from his athletes.

Procedural-based medicine has for over a decade engaged with a similar approach for the improvement of operative skills. This approach i.e., “proficiency-based progression” (PBP) (8, 19, 30), pays similar attention to the exacting level of detail as in the AMG approach used by Brailsford. The supposition underpinning PBP training is that individuals who are good at performing a procedure attend to small and apparently inconsequential aspects of performance that in isolation appear unimportant. However, when these small and detailed aspects of performance are effected and chained together, then that individual performs considerably better than an individual who is at best average at performing the same procedure. The PBP approach is however considerably more systematic and scientific than the AMG approach. In PBP, the performance skills to be taught and acquired are derived from a detailed and systematic procedure characterization. During this characterization process (described here), the specific attributes of optimal and suboptimal performance are identified and operationally defined rather than described (8, 19). Furthermore, rather than assuming, that these attributes accurately and comprehensively characterize the skills or procedure in question, the characterization is subjected to detailed and scientific validation, initially through a Delphi panel (20) with peers and then through quantitative construct validity testing that is objective, transparent and fair (32, 40).

We believe that is this attention to detail and quality assurance which will impact patient outcomes. It is the explicit identification, robust validation, and proficiency benchmarking of the performance metrics in a PBP training program that will ensure a more standardized approach to MT training leading to the production of more homogeneous skill levels of trainees that positively impacts patient outcomes.

The way doctors are trained to perform interventional procedures is evolving from an apprenticeship-type model to something that is more scientific, systematic, and evidence-based. A proficiency-based progression approach to training is based on performance metrics that are derived from, agreed upon by, and benchmarked by experienced and practicing clinicians who are “good” at performing the procedure. Physics-based virtual reality simulation training with the exact same procedure devices affords trainees the opportunity to acquire a very high level of procedure skill outside of the intervention suite and operating room, rather than training on real patients at the beginning of their learning curve in “apprentice-style training”. Evidence from prospective, randomized, and blinded studies have shown that simulation training without detailed performance metrics for formative feedback to the trainee and proficiency benchmarks is no better than an interesting educational experience. These performance benefits of proficiency-based progression training are too substantial to ignore. The bottom line of PBP training is that the performance level of the trainee must be known at the end of training. Furthermore, the trainee should only be allowed to proceed to perform on real patients when they have demonstrated the necessary proficiency benchmarks based on the performance of experienced operators/interventionalist.

## Conclusions

The PBP approach has been validated and proven beneficial across multiple medical disciplines. Mechanical thrombectomy can no doubt be a beneficiary too, with application across the multidisciplinary team including neurologists and anesthetists. We set the PBP approach in mechanical thrombectomy framed around the physics-based virtual reality simulation, as the successful and proficiently performed mechanical recanalization is the core of the treatment. Without recanalization, any additional improvements in the pre and post-angiography setting are rather pointless. But not only in the spirit of the Brailsford AMG approach, but PBP in thrombectomy also needs to incorporate the stroke treatment pathways before and after the Angio Suite to further reduce poor outcomes and improve the overall benefit of mechanical thrombectomy.

Another area that offers potential for additional study includes the use of this methodology for skill retention in procedures that may be infrequently performed. Intuitively there may be benefits here not just for trainees or clinicians early in their careers but also for experts. Incorporating a PBP refresher approach for a procedure that may only be performed on a handful of occasions per year could potentially be of great value and interest.

## Data availability statement

The original contributions presented in the study are included in the article/supplementary material, further inquiries can be directed to the corresponding author/s.

## Author contributions

MH, RC, TL, and AG contributed to conception and design of the study. AG, RC, and MH composed the first draft of the manuscript. All authors contributed to manuscript revision, read, and approved the submitted version.

## Conflict of Interest

The authors declare that the research was conducted in the absence of any commercial or financial relationships that could be construed as a potential conflict of interest. The reviewer ST declared a shared affiliation with one of the author, TL, to the handling editor at time of review.

## Publisher's Note

All claims expressed in this article are solely those of the authors and do not necessarily represent those of their affiliated organizations, or those of the publisher, the editors and the reviewers. Any product that may be evaluated in this article, or claim that may be made by its manufacturer, is not guaranteed or endorsed by the publisher.

## References

[1] Cates CU. Virtual reality simulation in carotid stenting: a new paradigm for procedural training. Nat Clin Pract Cardiovasc Med. (2007) 4:174–75. 10.1038/ncpcardio0837 17380164

[2] Cuschieri A. Whither minimal access surgery: tribulations and expectations. Am J Surg. (1995) 169:9–19. 10.1016/S0002-9610(99)80104-4 7818004

[3] Bergman RM Nerem RM. The cardiovascular technology industry: past, present, and future. Cardiovasc Eng Technol. (2010) 1:4–9. 10.1007/s13239-010-0010-x

[4] Leon MB Smith CR Mack M Miller DC Moses JW Svensson LG. Transcatheter aortic-valve implantation for aortic stenosis in patients who cannot undergo surgery. N Engl J Med. (2010) 363:1597–607. 10.1056/NEJMoa1008232 20961243

[5] Crossley R Liebig T Holtmannspoetter M Lindkvist J Henn P Lonn L. Validation studies of virtual reality simulation performance metrics for mechanical thrombectomy in ischemic stroke. J Neurointervent Surg. (2019) 11:775–80. 10.1136/neurintsurg-2018-014510 30655360 PMC6703121

[6] Crossley R Liebig T Holtmannspoetter M Lindkvist J Henn P Lonn L. Metric-based virtual reality simulation – a paradigm shift in training for severe stroke'. Stroke. (2018) 49:e239–e42. 10.1161/STROKEAHA 29866758

[7] Gallagher AG Cates CU. Virtual reality training for the operating room and cardiac catheterisation laboratory. Lancet. (2004) 364:1538–40. 10.1016/S0140-6736(04)17278-4 15500900

[8] Gallagher AG Ritter EM Champion H Higgins G Fried MP Moses G. Virtual reality simulation for the operating room: proficiency-based training as a paradigm shift in surgical skills training. Ann Surg. (2005) 241:364–72. 10.1097/01.sla.0000151982.85062.80 15650649 PMC1356924

[9] Institute Institute of Medicine Committee Committee on the Governance and Financing of Graduate Medical Education. Graduate Medical Education That Meets the Nation's Health Needs. Washington, DC: Institute of Medicine (2014). 25340242

[10] Ericsson KA Krampe RT Tesch-Römer C. The role of deliberate practice in the acquisition of expert performance. Psychol Rev. (1993) 100:363–406. 10.1037/0033-295X.100.3.363

[11] Seymour NE Gallagher AG Roman SA O'Brien MK Bansal VK Andersen DK. Virtual reality training improves operating room performance: results of a randomized, double-blinded study. Ann Surg. (2002) 236:458–63; discussion 63–4. 10.1097/00000658-200210000-00008 12368674 PMC1422600

[12] Ahlberg G Enochsson L Gallagher AG Hedman L Hogman C McClusky DA 3rd. Proficiency-based virtual reality training significantly reduces the error rate for residents during their first 10 laparoscopic cholecystectomies. Am J Surg. (2007) 193:797–804. 10.1016/j.amjsurg.2006.06.050 17512301

[13] Van Sickle KR Ritter EM Baghai M Goldenberg AE Huang IP Gallagher AG. Prospective, randomized, double-blind trial of curriculum-based training for intracorporeal suturing and knot tying. J Am Coll Surg. (2008) 207:560–8. 10.1016/j.jamcollsurg.2008.05.007 18926460

[14] Mazzone E Puliatti S Amato M Bunting B Rocco B Montorsi F. A systematic review and meta-analysis on the impact of proficiency-based progression simulation training on performance outcomes. Ann Surg. (2021) 274:281–9. 10.1097/SLA.0000000000004650 33630473

[15] Gallagher AG Cates CU. Approval of virtual reality training for carotid stenting: what this means for procedural-based medicine. J Am Med Assoc. (2004) 292:3024–26. 10.1001/jama.292.24.3024 15613672

[16] Food and Drug Administration. Draft Guidance for Industry and Food and Drug Administration Staff; Applying Human Factors and Usability Engineering to Optimize Medical Device Design. Silver Spring, MD: Department of Health and Human Services, Center for Devices and Radiological Health (2011).

[17] The American Board of Surgery. General Surgery Qualifying Examination-Overview Philadelphia. (2010). Available from: http://home.absurgery.org/default.jsp?certgsqe (accessed November 22, 2010).

[18] Zendejas B Brydges R Hamstra SJ Cook DA. State of the evidence on simulation-based training for laparoscopic surgery: a systematic review. Ann Surg. (2013) 257:586–93. 10.1097/SLA.0b013e318288c40b 23407298

[19] Gallagher AG O'Sullivan GC. Fundamentals of Surgical Simulation; Principles & Practices. London: Springer Verlag (2011).

[20] Angelo RL Ryu RK Pedowitz RA Gallagher AG. Metric development for an arthroscopic Bankart procedure: assessment of face and content validity. Arthroscopy. (2015) 31:1430–40. 10.1016/j.arthro.2015.04.093 26239785

[21] Angelo RL Ryu RK Pedowitz RA Beach W Burns J Dodds J. A proficiency-based progression training curriculum coupled with a model simulator results in the acquisition of a superior arthroscopic Bankart skill set. Arthroscopy. (2015) 31:1854–71. 10.1016/j.arthro.2015.07.001 26341047

[22] Satava RM Stefanidis D Levy JS Smith R Martin JR Monfared S. Proving the effectiveness of the fundamentals of robotic surgery (FRS) skills curriculum: a single-blinded, multispecialty, multi-institutional randomized control trial. Ann Surg. (2020) 272:384–92. 10.1097/SLA.0000000000003220 32675553

[23] Cates CU Lönn L Gallagher AG. Prospective, randomised and blinded comparison of proficiency-based progression full-physics virtual reality simulator training versus invasive vascular experience for learning carotid artery angiography by very experienced operators. BMJ Simul Technol Enhanc Learn. (2016) 2:1–5. 10.1136/bmjstel-2015-000090 35516451 PMC8936583

[24] Ahmed OM. D O'Donnell BD, Gallagher AG, Shorten GD. Development of performance and error metrics for ultrasound-guided axillary brachial plexus block. Adv Med Educ Pract. (2017) 8:257–63. 10.2147/AMEP.S128963 28435344 PMC5388285

[25] Ahmed OMA Niessen T O'Donnell BD Gallagher AG Breslin DS DunnGalvin A. The effect of metrics-based feedback on acquisition of sonographic skills relevant to performance of ultrasound-guided axillary brachial plexus block. Anaesthesia. (2017) 72:1117–24. 10.1111/anae.13968 28741649

[26] Ahmed OM O'Donnell BD Gallagher AG Breslin DS Nix CM Shorten GD. Construct validity of a novel assessment tool for ultrasound-guided axillary brachial plexus block. Anaesthesia. (2016) 71:1324–31. 10.1111/anae.13572 27634361

[27] Breen D O'Brien S McCarthy N Gallagher A Walshe N. Effect of a proficiency-based progression simulation programme on clinical communication for the deteriorating patient: a randomised controlled trial. BMJ Open. (2019) 9:e025992. 10.1136/bmjopen-2018-025992 31289064 PMC6629454

[28] Kallidaikurichi Srinivasan K Gallagher A O'Brien N Vinod Sudir Nick Barrett. Proficiency-based progression training: an ‘end to end'model for decreasing error applied to achievement of effective epidural analgesia during labour: a randomised control study. BMJ Open. (2018) 8:e020099. 10.1136/bmjopen-2017-020099 30327396 PMC6194403

[29] Mascheroni J Mont L Stockburger M Patwala A Retzlaff H Gallagher AG. International expert consensus on a scientific approach to training novice cardiac resynchronization therapy implanters using performance quality metrics. Int J Cardiol. (2019) 289:63–9. 10.1016/j.ijcard.2019.04.036 31088758

[30] Gallagher A. Metric-based simulation training to proficiency in medical education:-What it is and how to do it. Ulster Med J. (2012) 81:107–13. 23620606 PMC3632817

[31] Kojima K Graves M Taha W Cunningham M Joeris A Gallagher AG. AO international consensus panel for metrics on a closed reduction and fixation of a 31A2 pertrochanteric fracture. Injury. (2018) 49:2227–33. 10.1016/j.injury.2018.09.019 30268512

[32] Angelo RL Ryu RK Pedowitz RA Gallagher AG. The Bankart performance metrics combined with a cadaveric shoulder create a precise and accurate assessment tool for measuring surgeon skill. Arthroscopy. (2015) 31:1655–70. 10.1016/j.arthro.2015.05.006 26238730

[33] Neary PC Boyle E Delaney CP Senagore AJ Keane FB Gallagher AG. Construct validation of a novel hybrid virtual-reality simulator for training and assessing laparoscopic colectomy; results from the first course for experienced senior laparoscopic surgeons. Surg Endosc. (2008) 22:2301–9. 10.1007/s00464-008-9900-5 18553207

[34] Angelo RL St Pierre P Tauro J Gallagher AG Shoulder PBP Instructional Faculty. A proficiency-based progression simulation training curriculum to acquire the skills needed in performing arthroscopic Bankart and rotator cuff repairs—implementation and impact. Arthroscopy. (2021) 37:1099–106. e5. 10.1016/j.arthro.2020.11.040 33359814

[35] Palter VN Grantcharov T Harvey A Macrae HM. *Ex vivo* technical skills training transfers to the operating room and enhances cognitive learning: a randomized controlled trial. Ann Surg. (2011) 253:886–89. 10.1097/SLA.0b013e31821263ec 21394017

[36] Kilroy DA. Competency in the new language of medical education. Br Med J. (2009) 26:3–6. 10.1136/emj.2008.057935 19104084

[37] Birkmeyer JD Finks JF O'Reilly A Oerline M Carlin AM Nunn AR. Michigan bariatric surgery collaborative. Surgical skill and complication rates after bariatric surgery. N Engl J Med. (2013) 369:1434–42. 10.1056/NEJMsa1300625 24106936

[38] Khatri P Yeatts SD Mazighi M Broderick JP Liebeskind DS Demchuk AM. Time to angiographic reperfusion and clinical outcome after acute ischaemic stroke: an analysis of data from the Interventional Management of Stroke (IMS III) phase 3 trial. Lancet Neurol. (2014) 13:567–74. 10.1016/S1474-4422(14)70066-3 24784550 PMC4174410

[39] Ajmi SC Advani R Fjetland L Kurz KD Lindner T Qvindesland SA. Reducing door-to-needle times in stroke thrombolysis to 13 min through protocol revision and simulation training: a quality improvement project in a Norwegian stroke centre. BMJ Qual Saf. (2019) 28:939–48. 10.1136/bmjqs-2018-009117 31256015

[40] Angelo RL Pedowitz RA Ryu RK Gallagher AG. The Bankart performance metrics combined with a shoulder model simulator create a precise and accurate training tool for measuring surgeon skill. Arthroscopy. (2015) 31:1639–54. 10.1016/j.arthro.2015.04.092 26129726

